# Preparing for Ebola, the experiences of a national training team (Ghana)

**DOI:** 10.11694/pamj.supp.2015.22.1.6320

**Published:** 2015-10-10

**Authors:** Margaret Lartey, Peter Puplampu, Nana Ayegua Hagan Seneadza, Joseph Oliver-Commey, Serwah Amoah, Sally-Ann Ohene

**Affiliations:** 1 Department of Medicine, University of Ghana School of Medicine and Dentistry, Ghana; 2 Department of Medicine, Korle Bu Teaching Hospital, Ghana; 3 Public Health Department, Korle Bu Teaching Hospital, Ghana; 4 World Health Organization (WHO) Country Office, Ghana

**Keywords:** Ebola, African nations, WHO, MSF

## Commentary

The Ebola Virus Disease (EVD), a severe disease often associated with a high fatality rate, is caused by strains of the Ebola virus belonging to the family Filoviridae [1]. In March 2014, the World Health Organization was notified of an outbreak of the Ebola Virus Disease (EVD), in Guinea [2]. The outbreak subsequently spread to affect neighboring Liberia and Sierra Leone. For the first time in the history of the disease, international spread by air and land travel to Nigeria, USA, Spain, the UK, Senegal and Mali occurred [3]. Declared a Public Health Emergency of International concern by the WHO in August 2014 [4] and described by the Director-General of the WHO, Margaret Chan as the largest, most complex outbreak [5], the Ebola virus disease has so far affected 21,724 people with 8,641 deaths as at 21st January, 2015 [3]. The persistent spread of the disease in the worst affected countries and the continued risk of spread internationally, resulted in several countries scrambling to get prepared to detect and contain cases in the event of an outbreak.

Ghana's approach to the preparedness against Ebola virus disease (EVD) was multifaceted with training of health workers being a key aspect. To date, several categories of health workers have been trained in the spectrum of preparedness activities ranging from surveillance to case management. This paper highlights some of the experiences of the trainers in preparing the country to respond adequately to an outbreak. Ghana's Ebola Preparedness Plan was developed by the Ministry of Health and its partners [6]. The plan recommended building the capacity of all ten regional hospitals and teaching hospitals to manage EVD cases. In addition 3 National Ebola treatment centers (ETC) were to be set up to cater for the Northern, middle and Southern zones of the country. The initial set of trainings targeted teams that would work in the ETCs in addition to some key institutions. Given the unpredictability of where the first Ebola case would emerge and the fact that most health workers had no prior experience with the management of EVD, there was urgency for comprehensive training to quickly cover these designated facilities. In response to the epidemic, WHO-AFRO office invited teams from countries not yet affected by Ebola for training on Emergency Preparedness and Response to EVD in Brazzaville from 26-28th August 2014. The general objective was to ensure country preparedness to handle a potential Ebola Virus Disease (EVD) outbreak. A team of five (5) from Ghana made up of two clinicians, two epidemiologists and one health promotion officer participated in this training.

Among other objectives, the training sought to update participants on the ongoing EVD outbreak, orient them on the available tools, guidelines and strategies for prevention, surveillance, case management and control of EVD, and review current country preparedness and response plans in line with recent WHO recommendations on EVD. Coincidentally, in the same week of the Brazzaville training, Médecins Sans Frontières (MSF) organized a training in Brussels for experienced doctors, nurses and water, hygiene and sanitation (WHS) specialists recruited for the first time into a program for fighting an Ebola outbreak in affected countries. The purpose was to ensure that all trained personnel had sufficient knowledge of the disease and its transmission routes and were able to adopt safe behavior and work efficiently in an Ebola Treatment Center (ETC). Key aspects of the training included a description of the ETC's organization, its goals and its operating principles, the patient and staff flows and circuits, infection-control activities and measures and the protocols and measures for protecting staff, especially PPE. The training also focused on the importance of recognizing the psychosocial dimension in treating the disease for patients, their families and the staff to reduce the impact of stress, fear and stigma. The Ghana Embassy in Belgium in collaboration with the Ghana Health Service (GHS) supported the participation of an infectious disease (ID) physician and nurse in the MSF training in Brussels. Thus the Brazzaville and Brussels trainings paved the way for the setting up of a national team of 7 trainers from academia, public sector and the World Health Organization to lead that aspect of the response. These further expanded their net to include 4 more persons and together drew up a curriculum that was used to train other health workers with the necessary knowledge and skills to manage EVD across the country.

### Outcome

A total of ten training workshops were held over a period of 4 months spanning September to December 2014 with the number of participants per session ranging from 25 to 110. Overall, 571 health personnel consisting of doctors, nurses, pharmacy and laboratory staff, mortuary personnel, community health, disease control and surveillance officers, ambulance technicians and security personnel were trained to train others or offer service, Table 1. The training of health workers from the nation's premier teaching hospital, the 37 Military Hospitals (37MH), both located in Accra kick-started the training sessions. The convenience of being able to quickly mobilize staff from these two facilities and the wide spectrum of patients catered for by them was the rational that paved the way for the “pioneer class” to be mobilized from these hospitals. A first batch of 25 environmental officers from the Metropolitan Assembly was trained as the burial team that could be called upon to conduct safe burials. In all the trainings, heavy emphasis was placed on infection prevention and control measures. The subsequent trainings, each held over 3 days were conducted for teams from various facilities and institutions and were batched to accommodate as many people as feasible given the pressure to build capacity widely. The third day of the training was used to improve on the wearing and removal of personal protective equipment and other hygienic practices like disinfection. It was also used for return demonstration by participants who were going to cascade the training in their respective regions and institutions. In addition to public facilities, training was conducted for private, faith based, police facilities and the ambulance services. Other partners organized training for laboratory technologists, customs and other port officials. The distribution of these trainings is shown in Table 1.

**Table 1:** Nationwide Ebola preparedness trainings (Ghana)

Date	Region/Institution of Participants	Number	Training venue
September 2014	37 Military Hospital and Korle-Bu Teaching Hospital.	60	Accra, Greater Accra Region
September 2014	Accra Metropolitan Authority	25	Accra, Greater Accra Region
September - October 2014	Ashanti Regional Hospital, Komfo Anokye Teaching Hospital, Ashanti Region ETC	71	Kumasi, Ashanti Region
October 2014	Greater Accra Regional Hospital, Greater Accra ETC, Christian Health Association of Ghana	110	Accra, Greater Accra Region
October 2014	Private Health Sector	25	Accra, Greater Accra Region
November 2014	Northern Regional Hospital, Tamale Teaching Hospital, Northern Region ETC	72	Tamale, Northern Region
November 2014	Upper East and Upper West Regional Hospitals	42	Tamale, Northern Region
November 2014	Central and Western Regional Hospitals, Cape Coast Teaching Hospital	64	Cape Coast, Central Region
November 2014	Brong Ahafo, Eastern and Volta Regional Hospitals	54	Koforidua, Eastern Region
December 2014	Police Hospital, Accra Psychiatric Hospital,	48	Accra, Greater Accra Region
**Total**		**571**	

### Observations and Lessons learned

The initiation of the training was generally met with relief as health workers had heard and seen the devastating effects of Ebola in the media. In the earlier trainings, there was some discontent among some participants as they had been selected to attend and were not there by choice. This may have been due to concerns and fear of being expected to be called to manage cases in the event of an outbreak. As time went on however, perhaps recognizing the benefits of being trained, participants came willingly for subsequent trainings while others also volunteered to be trained.

#### Commitment by Training Team

The desire and passion to prevent an outbreak of Ebola motivated the team to work together effectively showing commitment by regular meetings, updating training material, training at weekends and night hours while contributing to production of information, education and communication materials in their spare time. The training team had a lot of support from the WHO Country Representative, the Director General of the GHS, the Ebola Incident Commander and their respective teams.

### Team Approach

The trainers advocated for a team approach to training where members of the team could be called to respond to a probable case. This team included physicians, nurses, laboratory technologists, psychologists and counselors, epidemiologists, logisticians, social mobilisers, sanitary and burial teams among others. This initially did not seem acceptable to the managers of facilities or the health service and in the initial few cases, training was done for any mix of participants. Every training session was a new experience for the trainers as they had to be up to date with new definitions, changing epidemiology, updated responses and statistics as well as the level of response by the country. Each training session also led to suggestions from participants on better ways of doing things and these were adopted as part of the country response.

#### Confidence of Health Workers

It was observed that the fear and panic that characterized the beginning of training sessions soon resulted in increased confidence by the end of the sessions. The health workers had become more relaxed and were willing to train others in what they had learned.

### Challenges

#### Changing Case definitions

One of the challenges the team had to deal with was changes in definitions and differing definitions for probable case, suspected case and alert case. This was compounded by dissemination of local definitions without prior notice to training teams. This led to a lot of unnecessary arguments by participants who had read these on the internet or web sites of response organizations. A consensus meeting was held to synchronize definitions.

#### Unavailable Training Materials

Some of the WHO materials for training, such as standard operating procedures for safe burials and the design of an ETC were initially not available when the training started, thus requiring trainers to be innovative and use locally prepared ones.

#### Personal Protective Equipment (PPEs)

Some participants were just interested in PPEs, not realizing that they needed a holistic understanding and approach to the Ebola prevention and control. As the trainings continued a new challenge of non-standardized and multiplicity of PPEs emerged, as many of them were donations from different sources. Trainers would arrive at a venue only to realize that PPEs allocated were different and had to be donned and removed in a different order. Even though the trainers quickly adapted, pre prepared posters and standard operating procedures (SOPs) then became useless leaving gaps in the implementation of the wearing and removal of PPEs.

#### Territorial control

Unfortunately some health worker groups insisted on maintaining their territory, training independently of the others. Others were not prepared to adapt from their original job schedules to support the response. The trainers had to do a lot of advocacy on the effectiveness of team training in responding to the epidemic and the “all hands on deck approach” during the response. Some of this played out in poor coordination with some members of the team totally forgetting their parts in the first simulation done at the ETC.

#### Challenges with practical sessions and large numbers

One of the challenges with the training for preparedness was the inadequate time allotted for practical sessions. Though trainers tried to increase the time for practical sessions with subsequent training sessions, this was still insufficient. This was also compounded by the fact that even those who had the opportunity of wearing and removing PPEs, without constant practice soon forgot the steps involved. The large number of participants in some of the training sessions did not allow for adequate attention to be paid to individual needs. Some participants complained about the theoretical nature of the classroom style training and advocated for hands on training in the ETC to get for a feel of how real Ebola case management would be in practice. This was however not possible as the ETC was being constructed at the same time that the trainings were ongoing.

#### Lack of funds

Lack of inadequate funds was a major challenge as the trainers tried to scale up to train a team in each of the ten regions of the country. Training sessions were cancelled at the last minute resulting in changes to schedules. The lack of funds also reflected in the numbers of PPEs available for participants to try out as most were being reserved for the actual epidemic.

#### Poor Infection Prevention and Control (IPC) knowledge and Practice

Despite ongoing infection prevention and control programs in the health institutions, majority of the participants had either forgotten or did not practice basic infection prevention practices. The participants also complained of lack of basic facilities like running water, waste disposal containers and PPEs like gloves at their workplace. This resulted in a lot of the training time being dedicated to IPC, thus leaving limited time for the other aspects of the Ebola management.

#### Dissatisfaction with National Response

Trainers were wrongfully misconstrued as the National Ebola Task Force thus taking a backlash for what was considered inadequacies by government and the MOH. This included no clear incentive policy for health workers who would opt to work in the ETC, inadequate logistics, perceived inability of the government to purchase and allocate enough PPEs slow pace in building ETCs and the slow pace of training both the public and private sectors.

## Conclusion

With national and regional teams trained, Ghana is better positioned to rapidly detect and manage a case of Ebola, contain and limit its spread should it occur. The national trainers, having shared their experiences still recognize that there is still more ground to cover and continue to work with regions and facilities to achieve this. In addition there is the need for frequent simulations and dry runs so that personnel do not forget the skills they have acquired. Monitoring is also essential to ensure that trainees are putting into practice what they have learned as well as maintaining the standards of IPC in the health facilities to improve on our preparedness.
